# High level of venous thromboembolism in critically ill trauma patients despite early and well-driven thromboprophylaxis protocol

**DOI:** 10.1186/s13613-017-0315-0

**Published:** 2017-09-12

**Authors:** S. R. Hamada, C. Espina, T. Guedj, R. Buaron, A. Harrois, S. Figueiredo, J. Duranteau

**Affiliations:** 1 Department of Anaesthesia and Critical Care, AP-HP, Hôpital Bicêtre, Hôpitaux Universitaires Paris Sud, University Paris-Sud, 78 rue du Général Leclerc, 94275 Le Kremlin Bicêtre, France; 2 Department of Anaesthesia and Critical Care, AP-HP, Hôpital Cochin, Hôpitaux Universitaires Paris Centre, University Paris Descartes, 27 Rue du Faubourg Saint-Jacques, 75014 Paris, France; 3 Department of Radiology, Hôpital Bicêtre, Hôpitaux Universitaires Paris Sud, University Paris-Sud, 78 rue du Général Leclerc, 94275 Le Kremlin Bicêtre, France

**Keywords:** Severe trauma, Venous thromboembolism, Risk factors, Thromboprophylaxis, Duplex ultrasound

## Abstract

**Background:**

Venous thromboembolism (VTE) is one of the most common preventable causes of in-hospital death in trauma patients surviving their injuries. We assessed the prevalence, incidence and risk factors for deep venous thrombosis (DVT) and pulmonary embolism (PE) in critically ill trauma patients, in the setting of a mature and early mechanical and pharmacological thromboprophylaxis protocol.

**Methods:**

This was a prospective observational study on a cohort of patients from a surgical intensive care unit of a university level 1 trauma centre. We enrolled consecutive primary trauma patients expected to be in intensive care for ≥48 h. Thromboprophylaxis was protocol driven. DVT screening was performed by duplex ultrasound of upper and lower extremities within the first 48 h, between 5 and 7 days and then weekly until discharge. We recorded VTE risk factors at baseline and on each examination day. Independent risk factors were analysed using a multivariate logistic regression.

**Results:**

In 153 patients with a mean Injury Severity Score of 23 ± 12, the prevalence of VTE was 30.7%, 95 CI [23.7–38.8] (29.4% DVT and 4.6% PE). The incidence was 18%, 95 CI [14–24] patients-week. The median time of apparition of DVT was 6 days [1; 4]. The global protocol compliance was 77.8% with a median time of introduction of the pharmacological prophylaxis of 1 day [1; 2]. We identified four independent risk factors for VTE: central venous catheter (OR 4.39, 95 CI [1.1–29]), medullar injury (OR 5.59, 95 CI [1.7–12.9]), initial systolic arterial pressure <80 mmHg (OR 3.64, 95 CI [1.3–10.8]), and pelvic fracture (OR 3.04, 95 CI [1.2–7.9]).

**Conclusion:**

Despite a rigorous, protocol-driven thromboprophylaxis, critically ill trauma patients showed a high incidence of VTE. Further research is needed to tailor pharmacological prophylaxis and balance the risks and benefits.

**Electronic supplementary material:**

The online version of this article (doi:10.1186/s13613-017-0315-0) contains supplementary material, which is available to authorized users.

## Background

Venous thromboembolism (VTE), comprising deep venous thrombosis (DVT) and pulmonary embolism (PE), is a potentially life-threatening complication in severe and multiple trauma patients [1]. It is one of the most common preventable causes of in-hospital death in trauma patients surviving their injuries [2]. It remains a challenging issue for the clinician as the clinical clues are rough and found in less than 5% of patients [3, 4], and the treatment should be balanced between risks and benefits [5, 6].

A number of risk factors and injury patterns have been identified to predict the occurrence of VTE [7, 8]. From the endothelial damage [9], to the activation of the coagulation system enhanced by the inflammatory state in association with hypotension, immobilization, stasis [10–12], and procoagulant therapies [13, 14], the whole system is unbalanced towards a predominant prohaemostatic state that remains for several days. As a consequence, VTE prophylaxis has to be started early and should combine mechanical and chemical prophylaxis [15]. Some guidelines are available to help the clinicians, but the level of evidence remains fairly low [5, 8]. Recent literature nevertheless tends to push forward early chemical thromboprophylaxis, tempering the fears of haemorrhagic complications, especially in the setting of solid organ and brain injuries [16].

The aim of this study was to assess the epidemiology of DVT and associated PE by repeated compression duplex ultrasound (DUS), in severe trauma patients in the setting of a rigorous and early mechanical and pharmacological thromboprophylaxis protocol.

## Methods

This prospective, 1-year observational study took place between February 2015 and 2016 in Bicêtre hospital, an academic level 1 trauma centre on the southern edge of Paris, with a 28-bed surgical and trauma intensive care unit (ICU), which receives an average of 500 suspected severe trauma patients per year. The local institutional review board approved the study and waived the need for informed consent (“Comité de Protection des Personnes”, No. SC 14-019). Patients and families were informed of the protocol. All consecutive trauma patients directly admitted in the ICU trauma room, were assessed and included if they were more than 18 years old and were expected to be in ICU for more than 48 h. Patients transferred from the emergency department or from another hospital were not included.

### Protocol

The local thromboprophylaxis protocol is extensively described in Appendix 1. DVT screening and follow-up were performed by DUS within the first 48 h, between 5 and 7 days and then weekly until ICU discharge. Both upper and lower extremities were examined: the internal jugular, subclavian, axillary, common femoral, superficial femoral and popliteal veins. Common iliac, internal and external iliac veins were also evaluated when possible (if no gas screen impeding penetration of US). A high-frequency transducer (5–10 MHz) was mostly used for upper extremities, but a 2–5-MHz transducer could also be used for lower extremities and abdominal imaging if necessary (Vivid I, GEMS Ultrasound, Tirat Carmel, Israel). The criteria to consider the diagnosis of a DVT were: non-compressibility of the vein (partial or total), direct visualization of the thrombus in the vein lumen and the absence of coloured or continuous Doppler flow. Each identified thrombus was categorized as “non-occlusive/mural”, “floating” or “occlusive”. Pulmonary embolism was diagnosed on CT angiography according to clinical suspicion.

For the purpose of the study, two practitioners (SH and CE) already experienced in ultrasound (US) imaging, received a complementary training with a radiologist specialized in US vascular imaging (TG) (10 complete examinations combined with theory). Every DVT diagnosis by SH and CE was confirmed by the radiologist (TG) either by a direct control or through several sequential video recordings.

To overcome some limitations of DUS screening, we planned a blind proofreading focused on DVT and PE of all contrast CT scans (including the initial injury assessment whole body CT scan with a biphasic injection) undergone by all patients enrolled in the study. A single, independent radiologist (RB) blinded of any medical history performed all the CT scan reviews.

All data were collected prospectively (Appendix 2).

### Endpoints

The primary clinical endpoints were the prevalence and incidence of VTE (DUS diagnosed DVT and PE) in our cohort of trauma patients admitted in the surgical ICU.

The secondary endpoints were the calculation of the total prevalence of VTE with CT scan re-readings, the analysis of risk factors (among clinical factors, resuscitative strategies, transfusion and prophylaxis), morbidity and mortality analysis of VTE and early thromboprophylactic strategies.

### Statistical analysis

Quantitative data are expressed as mean ± standard deviation or median [quartile 1; 3] according to their distribution. Categorical data are expressed as numbers and percentages. Two groups were identified: patients with VTE (group VTE+) and patients without any VTE (group VTE−). Statistical tests used for the univariate analysis were Student’s *t* test, Mann–Whitney test, Chi-square or Fisher’s exact test according to their validity conditions. For incidence and timing calculation, we only considered the first episode of DVT or PE. The probability of developing a VTE was estimated using a Kaplan–Meier method. Patients who died of other cause than VTE and patient who were discharged from the ICU were censored in the analysis. A multiple logistic regression was performed according to the standards and respectfully of validity conditions, to identify independent risk factors for VTE. The calibration (Hosmer–Lemeshow statistic) and discrimination (area under the curve, AUC) were calculated, and the model internally validated using a bootstrap methodology [17]. All variables were tested for interactions and collinearity. The variables were chosen for their clinical relevance and their pragmatism for daily use, prohibiting any pre-existing clinical score to enter into the model. They were chosen a priori if they were associated with VTE on the univariate analysis with a p < 0.1 and then assessed by a forward stepwise analysis to preserve a number of event-to-variable ratio around 10. The missing data were <1%.

All statistical analysis was performed using R 3.2.1 software (http://www.R-project.org/) and using a *p* < 0.05 as significant.

## Results

**Fig. 1:**
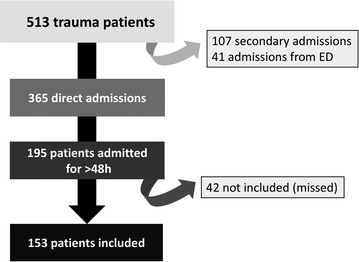
Flow chart of the study. Patients missed because length of stay was underestimated, or because the investigators of the study were not present during the first 48 h of inclusion

Over the 513 trauma patients admitted in 1 year, 153 were included in the analysis (flow chart displayed in Fig. 1). Demographic and clinical characteristics of the cohort are presented in Table 1.

**Table 1:** Demographic and clinical characteristics of included patients

Variable	All patients (*n* = 153)
Demographic characteristics	
Age (years)	44.6 ± 19.4
Sex (male)	72% (110)
BMI (kg/m^2^)	26.1 ± 6.5
Mechanism of injury	
MVA	26% (39)
Motorbike accident	21% (37)
Fall from high	25% (32)
Severity scores	
SOFA 24 h	6.4 ± 4.7
SAPS 2	34.9 ± 19.2
ISS	23.7 ± 12.7
Clinical presentation	
Initial GCS	12.4 ± 3.9
Vasopressor on day 1	22% (34)
RAP score	11.1 ± 5.6
ICU length of stay (days)	7 [4, 17]
Hospital length of stay (days)	26.5 ± 22. 9
Initiation of thromboprophylaxis (days)	2 ± 1
Mortality	11.8% (18)

Data expressed as mean ± SD or median [quartile 1; 3] according to distribution

Categorical variables expressed as percentage (*n*)

*BMI* body mass index, *MVA* motor vehicle accident, *ICU* intensive care unit, *GCS* Glasgow Coma Score, *SOFA* Sequential Organ Failure Assessment Score, *SAPS 2* Simplified Acute Physiology Score 2, *ISS* Injury Severity Score, *RAP score* Risk Assessment Profile

### Primary endpoint

A total of 59 DVTs were diagnosed with DUS and one with CT angiography in 46 patients (30.1%). Eleven patients developed more than one DVT (average 1.3, maximum 4). Seven patients presented a PE (4.6%), including one patient having no DVT found on control examinations. So the prevalence of at least one episode of VTE in our cohort over the studied period was 30.7%, CI 95 [24–39] (47 patients), with a calculated incidence of 18%, CI 95 [14–24] patients-week (18 patients over 100 are diagnosed with VTE over a 1-week ICU follow-up).

### Deep venous thrombosis (DVT)

**Fig. 2:**
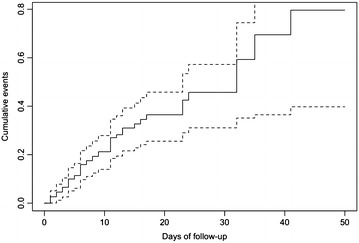
Kaplan–Meier curve of cumulative thromboembolic events in the cohort. We decided to include all patients presenting even with early diagnosis of DVT (within 48 h) as none of them had known risk factors (past medical history of DVT, or family history, cancer, obesity), nor any symptoms identified before admission. They were severely injured patients and had either extended vehicle extrication time or prolonged surgery with potentially compression mechanism

**Fig. 3:**
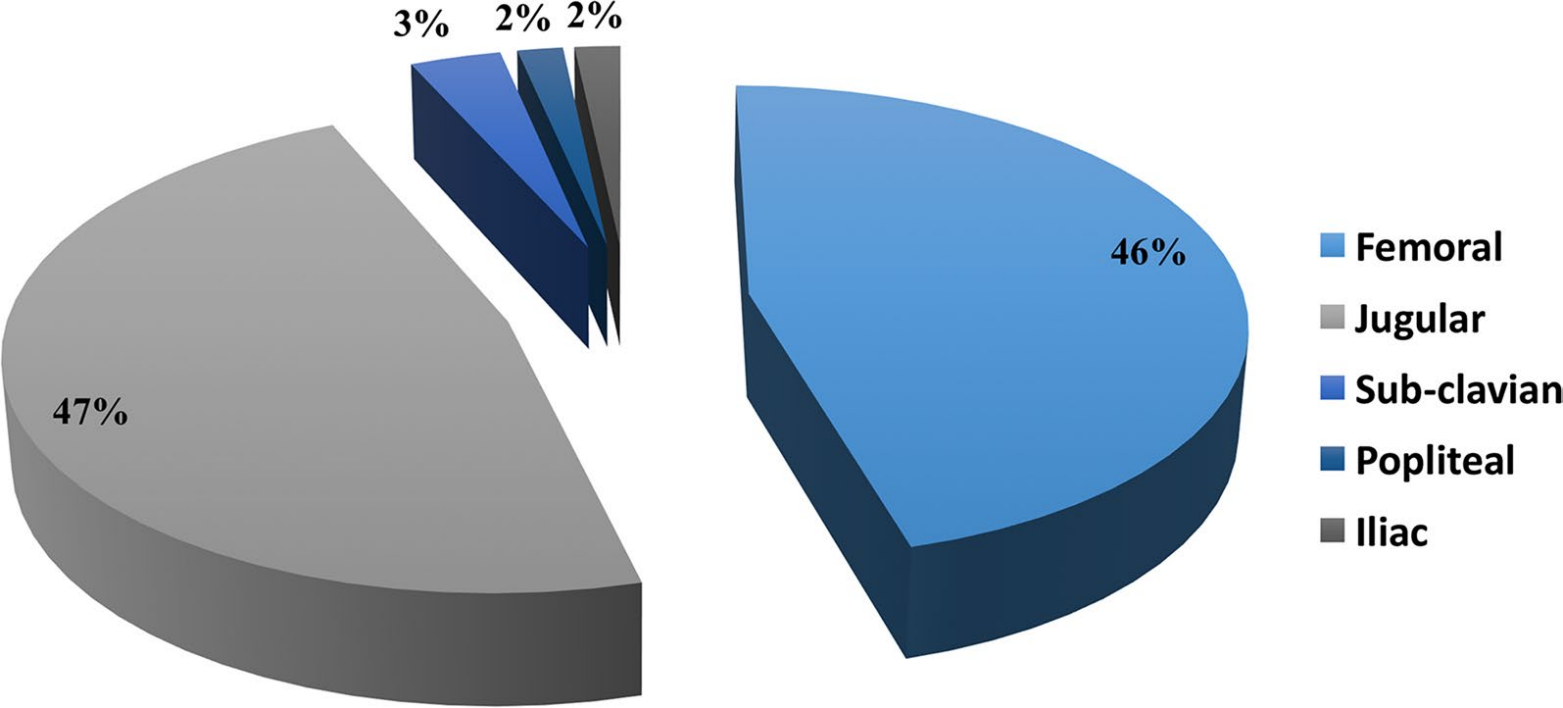
Anatomical situation of DVT diagnosed with duplex US examination (*n* = 59 in 45 patients). Femoral (*n* = 27), internal jugular (*n* = 29), subclavian (*n* = 2), popliteal (*n* = 1), external iliac (*n* = 1)

The majority of DVT was asymptomatic (56/60, 93%). The median time of apparition of DVT was 6 days [1; 4]. The Kaplan–Meier curve of probability of presenting a VTE is displayed in Fig. 2. Patients were followed during a median of 7 days [4; 17]. Eighteen DVT (40%) disappeared during their follow-up in a median time of 22 days [13; 25]. The diagnosed DVT was essentially located in the femoral and internal jugular vein as presented in Fig. 3. The types of thrombus found on DUS were categorized as “non-occlusive/mural” in 81.4% (*n* = 48), “floating” in 11.9% (*n* = 7) and “occlusive” in 6.7% (*n* = 4).

### Deep venous thrombosis and catheters

Three-fourth of DVTs (44/59) were diagnosed on the location of a central venous catheter (CVC), either on site or within the week following its withdrawal. The details of the association between DVT and CVC are presented in Table 2. Among the DVT unrelated to CVC (*n* = 16, 25.4%), seven were found in the femoral vein, eight in the internal jugular and one in the subclavian vein. The median time of apparition of DVT was 8 days [4; 12] when developing on CVC and 5.5 days [1.5; 12] when developing out of CVC.

**Table 2:** Relation between central venous catheter characteristics and DVT

Vein	Incidence of DVT (1000 catheter-days)	CVC (*n*) Patients (*n*)	DVT (*n*)	No. of days of catheterization	Length of catheterization (days)
Femoral	67.7	126/119	22	325	2 [1; 3]
I. jugular	47.5	53/47	21	442	8 [4.3; 12]
Subclavian	35.7	5/5	1	28	6 [4.8; 7.3]

No CVC (*n* = 29), 1 CVC (*n* = 73), 2 CVC (42), 3 CVC (*n* = 9)

Data expressed as median [quartile 1; 3]

*I. Jugular* internal jugular catheter, *DVT/1000 catheter-days* number of event/number of days of catheterization × 1000, *DVT* deep venous thrombosis, *CVC* central venous catheter

### Deep venous thrombosis and anticoagulants

The compliance with the local thromboprophylaxis protocol was observed in 119 patients (77.8%). The median time of introduction of the anticoagulant prophylaxis was 1 day [1; 2], with 80.4% (*n* = 123) of the patients receiving their first injection within the initial 48 h. Only four patients (2.6%) received the first injection after the 96th hour. LMW heparin accounted for 86.9% of the initial prescription (*n* = 133), when unfractionated heparin represented 11.8% (*n* = 18). Two patients did not receive any heparin, one died before the introduction (day 3), and the other one was an haemophiliac (who developed a DVT on day 8). There was no significant difference in the application of the protocol between the two groups.

In patients diagnosed with asymptomatic VTE, the introduction of a therapeutic dose of heparin was discussed on a case-by-case basis and 21 patients (44.7%) were curatively treated (outcome presented in Additional file 1: Table S1). We identified no complication related to pharmacological thromboprophylaxis. We did not find any worsening neither in clinically available monitoring of haemorrhage (tubes, drainage devices or wounds) nor in radiological imaging (volume of contusion or haematoma, haemorrhage relapse). Three patients benefited from a vena cava filter as they presented a contraindication for continuous anticoagulant therapy. Two still necessitated potentially haemorrhagic surgery, and one had complex pelvic fracture associated with a large cerebral contusion.

### CT scan review

A total of 205 CT scan angiograms were reviewed by the independent radiologist. Seven additional thromboembolic events were found in six patients: four segmental PE in patients who had no identified clinical symptom (outcome presented in Additional file 2: Table S2), and three DVT including one in internal jugular and two in common iliac veins. The overall prevalence was re-estimated at 34.6% (*n* = 53 patients).

### VTE and risk factors and outcome

Table 3 presents the results of the univariate analysis between the groups VTE + and VTE-. The multivariate analysis found no significant interactions among risk factors or risk factors and time. Four independent risk factors were identified: pelvic fracture (OR 3.04, 95% confidence interval (CI) [1.2–7.9]), CVC (OR 4.39, 95% CI [1.1–29]), medullar injury (OR 4.59, 95% CI [1.7–12.9]) and hypotension below 80 mmHg during initial management (OR 3.64, 95% CI [1.3–10.8]). The Hosmer–Lemeshow test showed a convenient fitting of the model with the observations (*p* = 0.94). The predictive performance showed an AUC of 0.74 [0.66–0.82]. Using bootstrap validation, the optimism-corrected ROC AUC was 0.73 [0.65–0.82], which represents a good predictive ability of the model in future patients [18].

**Table 3:** Univariate analysis of patient characteristics according to the occurrence of thromboembolic events

Variables	TEE+ (*n* = 47)	TEE− (*n* = 106)	*p*
Demographic characteristics			
Age (years)	46.8 ± 18.4	43.6 ± 19.8	0.3505
Sex (male)	80.9% (38)	67.9% (72)	0.6140
BMI (kg/m^2^)	26.6 ± 7.1	25.9 ± 6.2	0.5571
History of cancer	0% (0)	6 (5.7%)	0.2253
History of DVT	0% (0)	0% (0)	–
Injuries grading			
Initial GCS ≤ 8	29.8% (14)	17.9% (19)	0.1519
AIS thorax > 2	46.8% (22)	36.8% (39)	0.3230
AIS head > 2	40.4% (19)	43.4% (46)	0.8684
AIS abdomen > 2	27.6% (13)	17.9% (19)	0.2500
Pelvic fracture	29.8% (14)	12.3% (13)	0.0167
Lower limbs fracture	25.5% (12)	28.3% (30)	0.8746
Medullar injury	25.53% (12)	9.43% (10)	0.0179
Spine fracture	42.6% (20)	24.5% (26)	0.0402
Scores			
SAPS II	44.9 [38.7; 50.7]	30.4 [27.4; 34.1]	0.0001
ISS	28.9 [20; 34]	21.4 [13; 29]	0.0006
SOFA 24 h	8.3 [5; 11]	5.6 [1; 9.5]	0.0007
RAP score > 5	95.7% (45)	83% (88)	0.0582
RAP score > 10	70.2% (33)	39.6% (42)	0.0009
Clinical status			
SAP min (mmHg)	99 ± 28	111 ± 25	0.0083
Vasopressors	31.9% (15)	18.1% (19)	0.0594
Surgery > 2 h	46.8% (22)	30.2% (32)	0.0782
Central venous catheter	95.7% (45)	74.5% (79)	0.0041
Initial transfusion treatment (day 1)			
Transfusion > 4 RBC in 6 h	29.8% (14)	13.2% (14)	0.0264
Tranexamic acid	36.2% (17)	17.9% (19)	0.0246
Fibrinogen	31.9% (15)	13.2% (14)	0.0124
Thromboprophylactic procedure			
Compression stockings (absent)	8.5% (4)	9.5% (10)	0.9769
Pneumatic intermittent compression (no)	14.9% (7)	18.1% (19)	0.8877
Time before initiation of antithrombotic chemoprophylaxis	2 [1; 2]	1 [1; 2]	0.4533
Outcome			
ICU length of stay	18 [7; 32]	5.5 [3; 11]	<0.0001
Hospital length of stay	28 [15; 43]	17 [11; 28]	0.0027
Mortality	10.6% (5)	10.4% (11)	0.5976
Binarized characteristics			
ISS ≥ 16	87.2% (41)	69.6% (73)	0.0333
SAPS II ≥ 30	76.6% (36)	47.6% (49)	0.0016
SOFA 24 ≥ 5	80.9% (38)	50.9% (54)	0.0009
BMI ≥ 30 kg/m^2^	23.9% (11)	24.7% (21)	1
Age ≥ 50 years	40.4% (19)	34.6% (36)	0.614
SAP min ≤ 80 mmHg	26.1 (12)	7.8% (8)	0.0061
GCS ≤ 8	25.5% (12)	18.9% (20)	0.4718

Continuous data are expressed as mean ± SD or median [quartile 1; 3] according to their distribution. Categorical data are expressed as percentage (*n*)

*SAPS II* Simplified Acute Physiology Score, *SOFA à 24* *h* Sequential Organ Failure Assessment, *AIS* Abbreviated Injury Score, *ISS* Injury Severity Score, *RAP* Risk Assessment Profile, *DVT* deep venous thrombosis, *BMI* body mass index, *GCS* Glasgow Coma Score, *RBC* red blood cells, *systolic arterial pressure* minimal systolic arterial pressure recorded during transport, *ICU* intensive care unit

Regarding the clinical outcomes (bottom lines of Table 3), patients with VTE had a longer ICU length of stay (5.5 vs 18 days, *p* < 0.001) and longer hospital length of stay (17 vs 28 days, *p* < 0.003), but no difference was observed on mortality (14.9 vs 10.4%, ns).

## Discussion

This study demonstrates that in a population of critically ill trauma patients receiving early and protocolized thromboprophylaxis, the prevalence of VTE with a weekly DUS screening was as high as 30%.

### Epidemiology and prophylaxis in the literature

LMWH has been part of the standard of care for the prophylaxis of venous thromboembolism for more than 20 years. They have shown to be more effective than unfractionated heparin with an equal or even better level of safety. Several meta-analyses have confirmed a higher benefit/risk ratio. Major bleeding is less frequent. Heparin-induced thrombocytopenia is ten times less frequent with LMWH, and even in patients with renal insufficiency, they have demonstrated a higher ratio of efficacy/safety than unfractionated heparin [19]. In trauma patients, Geerts et al. [20] have shown that low molecular weight heparins were more effective than low-dose unfractionated heparin in preventing venous thromboembolism after major trauma.

The incidence in our study remains very high despite the protocol-driven early thromboprophylaxis (median time 1 day [1, 2]) and a satisfying global protocol compliance. Published incidences of VTE after trauma range from 4.6 to 28% [21–23] in patients receiving pharmacological and/or mechanical prophylaxis to 90% in patients receiving no pharmacological prophylaxis [24, 25]. In the largest reported venographic study, Geerts et al. [24] identified DVT in 58% of 349 trauma patients receiving no antithrombotic prophylaxis and undergoing contrast venography 1–3 weeks after admission. A recent Cochrane database review, including 16 studies (*n* = 3005 patients), concluded that any prophylaxis might reduce the risk of VTE: mechanical (RR 0.43, 95% CI [0.25–0.73]), pharmacological (RR 0.48, 95% CI [0.25–0.95]) or even better with both prophylaxis (RR 0.34, 95% CI [0.19–0.60]) [15]. The protocol we implemented in our unit combined all existing methods of prophylaxis, introduced as soon as possible for mechanical devices and specifying the earliest timing of introduction for the pharmacological prophylaxis. The median delay of introduction obtained in our cohort (1 day [1; 2]) is one of the shortest times we found in the literature. Hence, we can assume that VTE in our study was more due to a failure of the treatment than to a failure of treatment administration. In 2013, Gentile et al. [26] described a median delay of introduction of 13 days with 37% of trauma patients having no chemical prophylaxis. Nevertheless, the global trend is to start the antithrombotic prophylaxis more and more early, even in brain trauma patients [1, 16]. So far, providing LMWH within 36 h of trauma to all patients, including those with solid organ injuries or traumatic brain injuries, appears safe, effective and is recommended to reduce the incidence of DVT and VTE, without significant increase in bleeding [5, 27]. The optimal regimen dose is still debated: once or twice daily LMWH injections or twice daily unfractionated heparin. In a prospective cohort of 87 trauma patients, Ko et al. [28] showed that subprophylactic anti-Xa trough level was found in 73 of 87 patients (84%) with a twice daily 30-mg enoxaparin injection. This suggests that early monitoring and tailoring of anticoagulant prophylaxis in high-risk patients might be another research field to decrease the failure of VTE prevention.

### Duplex US and diagnosis

Duplex US is the modality of choice for the diagnosis and follow-up of symptomatic DVT [8], but its use in screening asymptomatic patients is still controversial. It remains an operator-dependant technique with varying reported performances between level I and level II studies (sensitivity 61–92%, respectively, and specificity 97–98%, respectively) [29]. Moreover, the use of US in screening asymptomatic patients is burdened by a low sensitivity when compared with venography [29, 30]. As a consequence, the incidence of DVT could just have been “underestimated” in our study. The double check allowed to minor the false positive, when false negative just relied on practitioners’ US performance. Moreover, in patients with overlying orthopaedic devices (external fixators, splints, bandages), only available areas were imaged. These local constraints, the low sensitivity of calf US vascular imaging [31] and the unknown significance of calf DVT explain why we intentionally focused our investigations on femoral and popliteal veins for the lower limb. Another hypothesis to explain the differences in incidence and performance reports could come from the non-uniform definition of thrombosis. A small mural thrombus on a CVC scar is probably different from an occlusive thrombus developing by itself. In our study, we decided to describe all thrombi corresponding to the protocol definition (“Methods” section). Nevertheless, on a pragmatic point of view, we addressed differently the different types of thrombus. We treated all floating and occlusive DVT without debating. Mural thrombi led to discuss the benefits/risks balance and really depended on the beliefs of the practitioner in charge.

### Risk factors

The risk factors identified in our study were in accordance with some of the risk factors identified in the trauma literature [7, 8, 23, 32]. The univariate analysis highlights the link between the severity of the patients and the exposition to transfusion and prothrombotic treatment and the risk of developing a VTE. Surprisingly, head AIS > 2 or GCS ≤ 8 was not associated with VTE. We might explain this observation by the fact that every patient, even those presenting severe traumatic brain injury, received a rather early chemical thromboprophylaxis, which is often not the case in other studies [1]. The individual risk factors (obesity, cancer, coagulation disorders or age) were not significantly associated with VTE because our cohort gathered mostly young patients, with little comorbidities. So if we had screened “high-risk patients” on the RAP score > 10, like Thorson et al. [23], we would have missed 14 patients (30%) what is, in an absolute point of view, quite consistent.

Our results also identified CVC as an independent risk factor for VTE (OR 4.1). This is a well-described risk factor in the trauma population, generally declined according to the insertion site. In our population, the femoral catheters were at highest risk of VTE (Table 3) as it was the emergency access (allowing simultaneous access of vein and artery for invasive arterial pressure monitoring), with delicate asepsis conditions and during the most prothrombotic period. The femoral site is known to be at higher risk of VTE in numerous studies (4, 37, 38), even in non-trauma patients [33].

### Strength

Concerning the method of selection of variables for the multiple logistic regression, we wanted a pragmatic, easy-to-use tool to identify patients at high risk of VTE. We excluded all pre-existing processed calculations (ISS, SAPS II, RAP, SOFA), and we focused on transfusion strategy, easy-to-identify injury pattern and resuscitation management. The CVC represents probably here a surrogate of global severity of the patient. As an inherent limit of this statistical tool, we could not prove causation but just identify association and adjust confounding factors one to the other. We could not test our model on a prospective validation cohort in this study.

### Limits

The limits of our study are inherent to its design. First, it is a prospective, single-centre, exploratory cohort study, which does not allow projecting the results over all trauma populations with different case mix and different prevention policies. Nevertheless, the baseline characteristics of our trauma population depict a cohort of classical, blunt, male, rather young trauma patients and our thromboprophylaxis protocol is detailed to allow sharing of information. Second, all the categories of DVT were pooled together, and occlusive thrombosis was the only symptomatic and was only 6.7. Third, this is a modest cohort of 153 patients that does not allow building up a robust predictive score, which was not the purpose of the study. Nevertheless, we tried to extract a signal for the clinicians to arise their awareness on the patients’ main risk of VTE. Fourth, and the most striking, is that the observed incidence is suspected to be rather “underestimated” by all the study bias. These statements highlight the failure of VTE prophylaxis in trauma patients, which is incentive to explore how to tailor adequate thromboprophylaxis in these patients [34, 35].

## Conclusion

 We found that the prevalence of VTE among trauma patients remaining in the ICU for more than 48 h and receiving protocolized early thromboprophylaxis was 31% (incidence 18% patients-week). Main identified independent risk factors were CVC, pelvic fracture, medullar injury or hypotension <80 mmHg during initial management. Further research is needed to determine whether a more intensified thromboprophylaxis regime would be safe and effective in severe trauma patients.

### Additional files

**Additional file 1: Table S1.** Outcome of patients who received a curative anticoagulation for a thromboembolic event secondary to trauma. DVT: deep venous thrombosis, PE: pulmonary embolism, CACT: curative anticoagulant therapy, ICU: intensive care unit, LOS: length of stay, ISS: Injury Severity Score.

**Additional file 2: Table S2.** Outcome of patients being diagnosed a posteriori with pulmonary embolism. LOS: length of stay, ISS: Injury Severity Score.

## Appendix 1: Venous thromboembolism prophylaxis protocol

- All patients receive elastic stockings and tight-and-leg intermittent pneumatic compression device as soon as possible after the second survey (excluding anatomical constraints).
- If patients have an immediate surgery or interventional radiology procedure, the intermittent pneumatic compression devices are set up post-operatively.
- The chemical prophylaxis by low-dose heparin, either unfractionated (5000 UI subcutaneous injection (SC)/12 h) or low molecular weight heparin (enoxaparin 40 mg SC/24 h), according to renal function, is started after 6 h post-operatively or following any invasive procedure in the absence of brain injury or latent haemorrhagic risk.
- For patients weighting more than 100 kg, heparin doses are increased to 5000 UI/8 h and enoxaparin 60 mg/24 h.
- For patients in haemorrhagic shock, heparin is started after bleeding control and restoration of biological haemostasis (platelets > 80.000/mm^3^, prothrombin time > 40%).
- For patients having a latent haemorrhagic risk (large solid organ contusion without embolization procedure, comminutive pelvic fracture, refractory epistaxis, etc.), the chemical prophylaxis is started after the first 24 h.
- For patients having spine or intracranial injuries, heparin is started 48 h after injury or neurosurgical intervention when a head CT scan shows stability of brain injuries and restoration of biological haemostasis (platelets > 100.000/mm^3^, prothrombin time > 60%).
- In case of an existing medical history of coagulopathy, the chemical prophylaxis is discussed on a case-by-case basis with haematologists.

## Appendix 2: List of data collected

- Demographic data including age, gender, weight and height for body mass index calculation (BMI kg/m^2^).
- Clinical data concerning comorbidities and medical history of thrombosis.
- Complete injury assessment (Abbreviated Injury Score of all body regions).
- Trauma management: injury mechanism, clinical data on scene and during transport (arterial pressure, Glasgow Coma Score, fluid load, vasopressor use), initial hospital coagulation and transfusion strategy (plasma, platelets, red blood cells, tranexamic acid, fibrinogen, activated factors), length of initial surgery.
- Severity (as day 1 scores): Injury Severity Score (ISS), Simplified Acute Physiologic Score (SAPS II), Sequential Organ Failure Assessment (SOFA) and Risk Assessment Profile (RAP) scores.
- Protocol compliance: elastic stockings (presence and timing), tight-and-leg intermittent pneumatic compression device (presence and timing).
- Modality of initial thromboprophylaxis (date of introduction of heparin, type of heparin, dose and respect of the protocol timing).
- All central venous catheters were identified and followed for insertion sites and length of maintenance.
- Clinical examination and coagulation screening tests (prothrombin time, fibrinogen, activated partial thromboplastin time and platelet count) on each day of DVT ultrasound screening.

## Abbreviations

DVT: deep venous thrombosis
VTE: venous thromboembolism
PE: pulmonary embolism
95 CI: 95% confidence interval
GCS: Glasgow Coma Scale
SAP: systolic arterial pressure
SOFA: Sequential Organ Failure Assessment
ISS: Injury Severity Score
RAP score: Risk Assessment Profile
CVC: central venous catheter
LMWH: low molecular weight heparin
CT: computed tomography
AIS: Abbreviated Injury Scale
SAPS II: Simplified Acute Physiology Score
US: ultrasound
DUS: venous duplex ultrasound
