# Supplementary material for: High level of venous thromboembolism in critically ill trauma patients despite early and well-driven thromboprophylaxis protocol
Source: Ann Intensive Care. 2017 Sep 12;7:97. doi: 10.1186/s13613-017-0315-0 (PMC5595705; doi:10.1186/s13613-017-0315-0)
Supplement: Supplementary file 1 — Additional file 1: Table S1. Outcome of patients who received a curative anticoagulation for a thromboembolic event secondary to trauma. DVT: deep venous thrombosis, PE: pulmonary embolism, CACT: curative anticoagulant therapy, ICU: intensive care unit, LOS: length of stay, ISS: Injury Severity Score. [file 13613_2017_315_MOESM1_ESM.docx]

| **Patient** | **DVT** | **PE** | **Age** | **Day of diagnosis** | **Type** | **Day CACT** | **Death** | **ICU LOS** | **Hospital LOS** | **ISS** |
| --- | --- | --- | --- | --- | --- | --- | --- | --- | --- | --- |
| CAC1 | Yes | No | 52 | 6 | Mural | 10 | No | 27 | 38 | 38 |
| CAC2 | Yes | No | 35 | 1 | Floating | 3 | No | 8 | 15 | 13 |
| CAC3 | Yes | No | 68 | 2 | Floating | 7 | No | 23 | 28 | 34 |
| CAC4 | Yes | No | 66 | 2 | Mural | 2 | Yes | 4 | 4 | 34 |
| CAC5 | Yes | No | 83 | 3 | Mural | 3 | Yes | 5 | 5 | 26 |
| CAC6 | Yes | Yes | 26 | 1 | Mural | 3 | No | 20 | 26 | 24 |
| CAC7 | Yes | Yes | 27 | 3 | Floating | 5 | No | 4 | 12 | 12 |
| CAC8 | Yes | No | 46 | 1 | Occlusive | 3 | No | 15 | 26 | 34 |
| CAC9 | Yes | No | 64 | 32 | Mural | 32 | No | 89 | 124 | 34 |
| CAC10 | Yes | No | 59 | 4 | Mural | 8 | No | 67 | 73 | 27 |
| CAC11 | Yes | Yes | 76 | 7 | Floating | 8 | No | 25 | 35 | 33 |
| CAC12 | Yes | No | 27 | 6 | Mural | 6 | No | 7 | 14 | 22 |
| CAC13 | Yes | No | 59 | 35 | Occlusive | 35 | No | 63 | 64 | 16 |
| CAC14 | Yes | Yes | 62 | 2 | Mural | 9 | No | 70 | 101 | 34 |
| CAC15 | Yes | Yes | 76 | 7 | Floating | 8 | No | 25 | 35 | 33 |
| CAC16 | Yes | Yes | 30 | 5 | Mural | 5 | No | 9 | 14 | 29 |
| CAC17 | Yes | No | 68 | 3 | Floating | 5 | No | 24 | 30 | 34 |
| CAC18 | Yes | No | 60 | 11 | Mural | 14 | No | 43 | 43 | 38 |
| CAC19 | Yes | No | 40 | 12 | Floating | 16 | Yes | 38 | 38 | 25 |
| CAC20 | Yes | No | 67 | 9 | Mural | 13 | Yes | 44 | 61 | 34 |
| CAC21 | Yes | Yes | 22 | 17 | Occlusive | 17 | No | 62 | 136 | 54 |
