# Supplementary material for: High level of venous thromboembolism in critically ill trauma patients despite early and well-driven thromboprophylaxis protocol
Source: Ann Intensive Care. 2017 Sep 12;7:97. doi: 10.1186/s13613-017-0315-0 (PMC5595705; doi:10.1186/s13613-017-0315-0)
Supplement: Supplementary file 2 — Additional file 2: Table S2. Outcome of patients being diagnosed a posteriori with pulmonary embolism. LOS: length of stay, ISS: Injury Severity Score. [file 13613_2017_315_MOESM2_ESM.docx]

|  | **Age** | **Death** | **ISS** | **Hospital LOS** |
| --- | --- | --- | --- | --- |
| **Patient 1** | 69 | 0 | 25 | 16 |
| **Patient 2** | 20 | 0 | 17 | 8 |
| **Patient 3** | 88 | 0 | 27 | 52 |
| **Patient 4** | 45 | 1 | 54 | 15 |
